# The Optimization of Breast Conservation

**DOI:** 10.4061/2011/456378

**Published:** 2011-12-12

**Authors:** William C. Dooley, Mo Keshtgar, Tibor Tot, Daigo Yamamoto, Mahmoud B. El-Tamer

**Affiliations:** ^1^The University of Oklahoma, Oklahoma City, OK 73104, USA; ^2^Royal Free and University College Medical School, University College London, London wc1 6BT, UK; ^3^Uppsala University, Osaka 570-8506, Sweden; ^4^Department of Surgery, Kansai Medical University, Japan; ^5^Memorial Sloan-Kettering Cancer Center, New York, NY 10065, USA

This issue includes information from the innovative research ongoing in breast cancer to increase our efficacy and therapeutic choices to adequately treat breast cancers with breast conservation. First a couple of articles address the biologic issues that form the basis of current therapies and how these may be improved with new biologic understandings. We are beginning to recognize that the breast is not one paired organ but two collections of intertwined ductal lobular trees. Most if not all breast cancers only involve a single ductal tree at the time of clinical detection. All the other ductal trees are at risk and may have both synchronous and metachronous lesions that can progress or regress based on biologic and environmental pressures. As we understand breast cancer biology better we may have opportunities to detect cancers earlier, prevent cancers, and optimize conservation with more accurate and precise treatment.

Oncoplastic surgery has given the prospect of breast conservation with reasonable cosmetic outcomes to more and more patients. It now becomes more important through biology and imaging that we accurately predict the extent of disease and treat with a single surgical intervention. Articles in this issue highlight these issues, challenges, and potential successful resolutions. It would seem now that 50–80% or more of stage 0–2 breast cancers could be treated equally as well through modern conservation techniques. 

One of the requirements for most patients now for breast conservation is radiation therapy. This has been historically very costly in both equipment and time commitment. New technologies and approaches are leading to much shorter treatment times and treatment volumes than the classic whole breast treated 5 days/week for 6–8 weeks. To make breast conservation more accessible in the less affluent parts of the world, we need short treatment times with minimal equipment and infrastructure investments. Several authors have presented data in this issue on evolving technologies including accelerated partial breast irradiation and targeted intraoperative radiotherapy. A single fraction of radiotherapy given during surgery directly to the tumor bed (intraoperative radiotherapy) avoids many of the prior problems. The rationale and level 1 evidence for the safety and efficacy of these approaches are reviewed and suggest that our ability to bring robust effective breast conservation irradiation to the entire world is soon going to be within our grasp.

The next two decades will see an explosion of breast cancer cases worldwide. Breast cancer becomes more common as countries gain in GDP (Gross Domestic Product). Rates for breast cancer in many parts of the world will reach that of Western Europe and North America. With this impeding public health problem, we need better screening, precise and cost effective treatment, and survivorship not unencumbered by complications and toxicities of our therapies. This issue brings data and ideas that offer a glimmer that breast conservation can become the most common treatment worldwide-not just in the affluent West.

*William C. Dooley* *William C. Dooley* *Mo Keshtgar* *Mo Keshtgar* *Tibor Tot* *Tibor Tot* *Daigo Yamamoto* *Daigo Yamamoto* *Mahmoud B. El-Tamer* *Mahmoud B. El-Tamer*

